# Stereotactic Body Radiation Therapy for Lung and Liver Oligometastases from Breast Cancer: Toxicity Data of a Prospective Non-Randomized Phase II Trial

**DOI:** 10.3390/curroncol29100621

**Published:** 2022-10-17

**Authors:** Davide Franceschini, Tiziana Comito, Anna Di Gallo, Veronica Vernier, Marco A. Marzo, Luciana Di Cristina, Beatrice Marini, Lorenzo Lo Faro, Sara Stefanini, Ruggero Spoto, Luca Dominici, Ciro Franzese, Marta Scorsetti

**Affiliations:** 1 Department of Radiotherapy and Radiosurgery, IRCCS Humanitas Research Hospital, Via Manzoni 56, 20089 Milan, Rozzano, Italy; 2 Department of Biomedical Sciences, Humanitas University, Via Rita Levi Montalcini 4, 20072 Milan, Pieve Emanuele, Italy

**Keywords:** stereotactic body radiotherapy, oligometastases, breast cancer, phase II, toxicity

## Abstract

Aims: We report the mature toxicity data of a phase II non-randomized trial on the use of SBRT for lung and liver oligometastases. Methods: Oligometastatic patients from breast cancer were treated with SBRT for up to five lung and/or liver lesions. Inclusion criteria were: age > 18 years, ECOG 0–2, diagnosis of breast cancer, less than five lung/liver lesions (with a maximum diameter <5 cm), metastatic disease confined to the lungs and liver or extrapulmonary or extrahepatic disease stable or responding to systemic therapy. Various dose–fractionation schedules were used. Then, a 4D-CT scan and FDG-CTPET were acquired for simulation and fused for target definition. Results: From 2015 to 2021, 64 patients and a total of 90 lesions were irradiated. Treatment was well tolerated, with no G 3–4 toxicities. No grade ≥3 toxicities were registered and the coprimary endpoint of the study was met. Median follow-up was 19.4 months (range 2.6–73.1). Conclusions: The co-primary endpoint of this phase II trial was met, showing excellent tolerability of SBRT for lung and liver oligometastatic in breast cancer patients. Until efficacy data will mature with longer follow-up, SBRT should be regarded as an opportunity for oligometastatic breast cancer patients.

## 1. Introduction

Breast cancer is the most common malignancy in women, accounting for almost one-third of all new cancer diagnoses. Breast cancer is the leading cause of cancer-related deaths among women aged 20 to 59 years, and it is the second most common cause of cancer mortality in women of all ages, following lung cancer [1]. Approximately, 5–10% of all breast cancer present with distant metastases at the time of diagnosis (“de novo” metastatic breast cancer), while 20% of patients with initially localized disease will develop metastases within 5 years of their initial diagnosis, despite treatment.

The term oligometastases derives from the ancient Greek ‘oligos’, meaning ‘few’, and ‘metastasis’, meaning ‘relocation’. The first time this concept was presented in the literature was in 1995, when Hellman S. and Weichselbaum R. proposed oligometastatic disease (OMD) as a distinct cancer state between locally confined and systemically metastasized disease [2]. About 20% of stage IV breast cancer patients can be considered oligometastatic at a certain point during their oncological history [3].

One important implication of the oligometastatic concept is that the local control (LC) of an oligometastatic disease (via surgical, radiotherapy, or thermo-ablative approaches) can have the potential to achieve disease cure and thus to prevent or delay cancer systemic spreading. Indeed, various phase II randomized trials recently confirmed the important role of local therapies (mainly Stereotactic Radiotherapy—SRT) in the management of these patients [4,5,6,7,8,9]. However, only in the SABR-COMET trial were breast cancer patients included. In the UK registry of oligometastatic patients recently published, although present, breast cancer patients represented only a minority of cases (5.5%) [10]. Two phase II non-randomized trials focused on breast cancer oligometastases; however, they both focused on a particular subset of patients: those affected by bone or bone and nodal metastases [11,12].

Prospective data on the treatment on visceral metastases are lacking. Therefore, in 2015, we started the study “Prospective Non-randomized Phase 2 Study on Stereotactic Body Radiation Therapy (SBRT) for Medically Inoperable Lung and Liver Oligometastases from Breast Cancer”, which was designed to evaluate the safety and efficacy of lung and liver SBRT in oligometastatic breast cancer patients deemed to be medically inoperable, using the VMAT RapidArc approach. The co-primary endpoints were local control (LC) rate and acute and late toxicity. The secondary endpoints were the evaluation of distant progression-free survival (DPFS) and overall survival (OS).

Here, we report the mature toxicity data of the trial.

## 2. Material and Methods

From November 2015 to October 2021, patients affected by lung and or liver oligometastases candidate to SBRT were enrolled in this phase II non-randomized trial (ClinicalTrials.gov Identifier: NCT02581670). The study was conducted with the approval of institutional review boards, and each patient signed an informed consent form.

To be included in the study, the patients had to be over 18 years old, with a good performance status, meaning a score from 0 to 2 on the Eastern Cooperative Oncology Group (ECOG) performance status scale, and they had to be free of life-threatening conditions. All patients received a diagnosis of breast cancer; liver and lung lesions had to be less than five with a maximum diameter of 5 cm; the metastatic disease had to be either confined to lungs and/or liver, with no extrapulmonary or extrahepatic disease, or if other metastatic sites were present, they had to be stable or responding to chemotherapy. Chemotherapy had to be completed at least 3 weeks before the beginning of treatment or started at least 2 weeks after radiotherapy. Systemic therapies such as hormonal therapies and immunotherapy were allowed during treatment. Both synchronous and metachronous metastases were accepted. Disease was considered synchronous when metastases occurred within 6 months from primary diagnosis. Lastly, a written informed consent was necessary.

Patients were instead excluded from the study in case of poor performance status, if pregnant or unable to give consent.

### 2.1. SBRT Procedure and Treatment Planning

The first step in the procedure was patient simulation. The patients were positioned supine, and thermoplastic masks were used as an immobilization system for the thoracic and abdominal region. For patients candidate to liver SBRT, an abdominal compressor device was used to reduce the liver motion related to the respiratory cycle. This allowed precisely reproducing the patient’s position during treatment delivery, thus reducing the minimum set-up errors.

A 4D-CT scan and FDG-CTPET were acquired for simulation and fused for target definition. In case of liver lesions, when deemed useful for contouring purposes and in the absence of contraindications, patients also performed abdominal MRI.

The internal target volume (ITV) was outlined by contouring the tumor (gross tumor volume, GTV) on all phases of the 4D-CT scan. The planning target volume (PTV) was obtained by adding a 5 mm margin to the ITV.

Special attention was given to organs at risk (OARs), meaning the critical structures that should be spared as much as possible of irradiation, which were outlined per each individual patient.

When irradiating liver lesions, the OARs included the liver free of disease, the kidneys, the spinal cord, the stomach, the duodenum, the chest wall and the bowel.

As far as lung lesions were concerned, the OARs were the lungs outside of the ITV, the spinal cord, the esophagus, the stomach, the hearth, the thoracic wall, the brachial plexus, the liver, and the trachea.

The specific dose constraints related to the OARs are summarized in the Table 1 and Table 2. In case of lung lesions, the dose constraints depended on the number of fractions.

All patients were treated with Volumetric Modulated Arc Therapy (VMAT) plans, which were optimized by inverse planning to ensure maximal dose conformity and rapid dose falloff toward critical structures. SBRT/VMAT was delivered with 6- or 10-MV photons, using modulated dynamic arcs. Dose was prescribed to target, ensuring that more than 98% of PTV received 95% of prescribed dose.

The suggested treatment schedule for liver lesions forecasted a standard dose of 75 Gy over 3 fractions, with 25 Gy per fraction. The dose could be decreased down to 3 levels, with the 1st level prescribing a total dose of 67.7 Gy, the 2nd level of 61.8 Gy and the 3rd level of 56.25 Gy; all of these doses were subdivided in 3 fractions.

As far as lung lesions were concerned, the recommended total dose was dependent on tumor size; 3 fractions were advised for tumors smaller than 1 cm, whereas for tumors 1 to 5 cm, a total dose of 60 Gy was proposed. In case of peripheral lung lesions, even the distance from the thoracic wall was taken into account to determine the number of fractions. In case of lesions closed to the chest wall, 4 or 5 fractions of 12 Gy each were chosen. In case of central lung lesions, 10 Gy × 5 fractions or 7.5 Gy × 8 fractions were considered.

Both in liver and lung lesions, the choice of total dose delivered fractionation was dependent on the constraints of healthy tissues.

During treatment, the position of all patients was assessed every session via image guidance through Cone Beam CT.

Corticosteroids were administered during SBRT if necessary and tapered as soon as possible.

### 2.2. Statistical Considerations

The aim of the study was to evaluate stereotactic radiation therapy schedules for liver and lung lesions in oligometastatic breast cancer in terms of safety and efficacy.

The primary endpoints of this clinical trial were to assess the LC rates of the irradiated lung and liver lesions and to monitor acute and late treatment-related lung and liver toxicities according to the National Cancer Institute Common Terminology Criteria and Adverse Events (CTCAE) version 4.0.

The secondary endpoints were the evaluation of progression-free survival (PFS) and overall survival (OS), the time to polyprogression and the time to start/change of systemic therapy.

This study adopted the A’hern design, which is a single-arm phase II study design whose aim is to assess the effects of a specific treatment on a binary endpoint at a fixed point in time.

According to the study design, it was expected that radiotherapy could increase the 2 years local control rate from 60% to 80%. In order to exclude a proportion of 60% at an error = 0.025, 1 side, 57 patients had to be entered and at least 42 patients in LC should be observed at 2-year follow-up in order to have a power of 90% under H1 hypothesis of 24 months LC proportion ≥80%.

With the same approach, for the other co-primary endpoint (severe toxicity, meaning grade 3 or higher), 58 patients needed to be enrolled, and severe toxicity had to be observed in not more than 10 patients in order to exclude a proportion of 30% severe toxicity at an error = 0.025, 1 side, and to have a power of 90% under H1 hypothesis <12% severe toxicity.

### 2.3. Follow-up

The patients were evaluated through history, performance status assessment, physical examination, blood tests and toxicity assessment 2 months after the end of treatment and then every 3 months for the first year and every 6 months from the second year up to five years.

Patients underwent CT scan within 2 months after SBRT and every 3 months thereafter, and FDG-CT PET 3 months and 1 year after radiation therapy.

Toxicities were graded according to the CTCAE version 4.0.

The response of target lesions was evaluated using the RECIST criteria.

## 3. Results

A total of 64 patients were enrolled in the study from 2015 to 2021. Patient and disease characteristics are summarized in Table 3.

The mean age was 61 years (ranging from 32 to 87 years) and the majority of patients (*n* = 40, 63%) had an ECOG performance status of 0. Ductal infiltrating carcinoma—or carcinoma of non-special type (NST)—was the most represented histology (*n* = 53, 83%), followed by lobular infiltrating carcinoma (*n* = 5, 8%), and mixed lobular and ductal histology (*n* = 2, 3%). Here, 70% (*n* = 45) and 56% (*n* = 36) of cases were positive for estrogen and progesterone receptors, respectively; HER2 was positive in 30% (*n* = 19) of cases; the mean Ki67 was 33% (ranging from 7% to 90%).

Only 9% (*n* = 6) of patients received neoadjuvant chemotherapy, while 94% (*n* = 60) underwent surgery; adjuvant chemotherapy was administered to 53% (*n* = 34) patients; and 61% (*n* = 39) of patients received adjuvant hormone therapy.

Metastatic disease and treatment features are summarized in Table 4.

The metastatic disease was synchronous in 23% (*n* = 15) of cases and metachronous in the rest, with a mean time from diagnosis to metastases of 1701.39 days (range 0 to 6514); the disease presented as oligometastatic only in 8% (*n* = 5) of cases. As far as treatments preceding SBRT, 8% (*n* = 5) did not receive any line of systemic therapy before radiotherapy, 36% (*n* = 23) received one line, 20% (*n* = 13) received two lines of therapy, and 36% (*n* = 23) received three or more lines of systemic therapy prior to SBRT. Only a minority of patients (*n* = 20, 31%) received other local ablative treatments (LATs) before SBRT.

The majority of patients (*n* = 23, 36%) were treated on induced oligoprogression, while 31% (*n* = 20) on induced oligopersistence, according to the ESTRO-EORTC classification [13].

Twenty-three (36%) patients were irradiated to the lung, forty (63%) were irradiated to the liver and one patient (2%) received radiotherapy to both organs. In most cases (*n* = 44, 69%), there was a single target lesion; 15 patients (23%) were treated for two lesions; while 3 and 4 lesions were irradiated, respectively, in four (6%) patients and one (2%) patient. The mean biologically effective dose (BED) was 140 (range 100 to 262.5).

In most cases, the metastatic disease was confined to the RT target (*n* = 41, 64%).

Concomitant systemic therapy was administered in 84% (*n* = 54) of patients, while 64% (*n* = 41) underwent some sort of systemic therapy after SBRT.

Among the 64 treated patients, a total of 20 (31.25%) experienced some type of toxicity (Table 5). Fifteen patients (23%) developed acute radiation-related toxicities, and in nine patients (14%), late toxicities were registered. No grade ≥3 toxicities were registered. Concerning acute toxicities, two patients (3%) developed a grade 2 toxicity (G2 nausea); the remaining acute toxicities that were recorded were grade 1 (G1 fatigue, nausea, fever, chest pain, abdominal pain and malaise). Late toxicities included G2 erythema (*n* = 1, 2%) and duodenal ulcer (*n* = 1, 2%), and G1 chest pain, cough, pneumonia, rib fracture, gastritis and gastrointestinal pain.

No severe toxicity was recorded, and the co-primary endpoint of the study was therefore met.

## 4. Discussion

We report the toxicity results of a phase II non-randomized trial evaluating the use of SRT for lung and liver oligometastases in breast cancer patients. The toxicity profile of SRT in our patients was excellent, and the trial met its co-primary endpoint, since no G3 or higher toxicity was recorded.

The safety profile of SBRT with modern technologies and a better understanding and definition of OARs dose constraints is nowadays well established. However, there are still some body sites requiring further research and attention due to the risk of severe toxicity. For lung SBRT, for instance, the ideal dose and fractionation schedule for central and ultracentral lesions is still to be defined. The seminal work by Timmerman and colleagues in 2006 [14] raised a significant alert about the use of three fractions SBRT in this area, which is due to excessive bronchial and vascular severe toxicity. This led to the definition of the so-called “no fly zone” to indicate an area located within 2 cm of the proximal bronchial tree, heart, great vessels, trachea, or other mediastinal structure where SBRT could not be delivered as safely as for peripheral nodules. However, in the subsequent years, retrospective and prospective studies validated the use of four, five or eight fractions schedules, showing equivalent efficacy and toxicity of three or four fractions SBRT for peripheral lesions [15,16]. On the contrary, the recently terminated HILUS trial showed a persisting issue of severe toxicity when treating ultracentral lung lesions with eight fractions SBRT. This was a phase II trial evaluating SBRT (7 Gy × 8 fractions) to lesions located close to the central airways. Despite the long fractionation schedule chosen, Grade 3 to 5 toxicity was noted in 22 patients, including 10 cases of treatment-related death (bronchopulmonary hemorrhage, n. 8; pneumonitis, n. 1; fistula, n. 1) [17]. In our trial, 24 patients received SBRT for 30 lung metastases. In case of central location (11 lesions), we chose 50 Gy in 5 fractions or 60 Gy in 8 fractions, prioritizing the OARs dose constraints in case of PTV overlap. Although limited by the small number of cases and the short follow-up time for the last enrolled patients, we did not observe significant toxicity in this subpopulation.

This result is in line with our previous experience of SBRT for central and ultracentral lung lesions; there, we recorded a limited grade ≥3 toxicity rate (5%) [18].

Furthermore, in our study, 31% of patients were treated for two or more lesions. The use of SBRT on multiple lesions was investigated in a phase 1 trial by Chmura et al., who were able to demonstrate the safety of SBRT for patients with three to four metastases or two metastases in close proximity [19]. They found that the SBRT dosing schemes developed for a single metastasis or primary tumor can be used to treat patients with multiple metastases with acceptable short-term toxic effects.

Historically, the role of radiotherapy in the treatment of liver tumors was limited by the risk of liver toxicity, particularly in terms of radiation-induced liver disease (RILD) and central hepatobiliary (cHB) toxicity. The most described form of liver toxicity is “classic” RILD, which is characterized by anicteric hepatomegaly and ascites. “Non-classic” RILD involves the elevation of liver transaminases more than five times the upper limit of normal reference or a decline of liver function [20]. The risk of RILD is proportional to the mean dose of radiation delivered to normal liver tissue. Therefore, it becomes feasible to safely treat hepatic lesions with high radiation doses by using SBRT, provided that the mean dose to normal liver can be kept well below the threshold above which severe RILD is observed. According to the studies by Schefter and Kavanagh [21], in our experience, it was shown to be safe to treat liver metastases with a prescription dose of 75 Gy in three fractions, as long at least 700 mL of normal liver received a <15 Gy.

Hepatobiliary toxicity is another form of liver toxicity that is apparently not induced by direct hepatocyte injury but rather caused by an insult to the central hepatobiliary tract (cHBT) [22]. Risk of central HB toxicity has previously been shown to have a dose-dependent relationship with the volume of the cHBT receiving a high dose of radiation. From 2017, Toesca et al. confirmed that high doses of radiation to the cHBT delivered by SBRT are associated with cHB toxicities in patients with primary liver cancer (cholangiocarcinoma and hepatocarcinoma) but not in patients with metastatic liver tumors [23]. In our experience, this suggestion was confirmed, considering that we have not found any cases of central biliary toxicity.

There are some limitations to acknowledge in our trial. The relatively low number of enrolled patients and the follow-up are still short for the latter enrolled patients, although they are enough for evaluating the pre-established toxicity endpoint of the study, analysis still requires caution. Patients will continue their follow up as scheduled not only for the evaluation of survival outcomes but also to detect further possible late toxicities. On the other side, the prospective nature of the trial and the well-described and a priori defined RT procedures represent the major strengths.

## 5. Conclusions

SBRT for the treatment of liver and lung oligometastases from breast cancer is safe and feasible. No severe toxicity was detected in this phase II trial, which is designed with co-primary endpoints of local control and safety. Longer follow-up is required to evaluate survival outcomes. Until that and considering the literature data already available, SBRT should be regarded as an opportunity for oligometastatic breast cancer patients.

## Figures and Tables

**Table 1:** Organs at risk’s dose constraints for liver lesions.

OARs	3 Fractions	6 Fractions
Liver (Disease free—cc)	≥700 cc < 15 Gy	≥700 cc < 21 Gy
Kidneys	D Mean < 12 Gy	D Mean < 12 Gy
Right kidney	D 65% < 15 Gy	D 65% < 15 Gy
Left kidney	D 30% < 15 Gy	D 30% < 15 Gy
Spinal cord	D Max < 18 Gy	D Max < 27 Gy
Stomach	D Max < 21 Gy	D Max < 36 Gy
Duodenum	D Max < 21 Gy	D Max < 36 Gy
Bowel	D Max < 21 Gy	D Max < 36 Gy
Chest wall	<30 cc < 30 Gy	<30 cc < 30 Gy

**Table 2:** Organs at risk’s dose constraints for lung lesions: ^1^ Mean Lung Dose.

OARs	3 Fractions	5 Fractions	8 Fractions
Lungs (minus ITV)	V5_Gy_ ≤ 20% V10_Gy_ ≤ 12% V20_Gy_ ≤ 10% MLD ^1^ ≤ 4 Gy	V5_Gy_ ≤ 30% V10_Gy_ ≤ 20% V20_Gy_ ≤ 10% MLD ≤ 4 Gy	V10_Gy_ ≤ 20% V15_Gy_ ≤ 37% MLD ≤ 7 Gy
Spinal cord	D Max < 18 Gy	D Max < 30 Gy	D Max < 32 Gy
Esophagus/stomach	D Max < 21 Gy	D Max < 30 Gy	D Max < 38.4 Gy D 5 cc < 23.7 Gy D 10 cc < 26.3 Gy
Heart	D Max < 30 Gy	D Max < 35 Gy	D Max ≤ 40 Gy D 15 cc < 34.4 Gy
Thoracic wall	D Max < 30 Gy	D Max < 32.5 Gy D 30 cc < 30 Gy	D Max < 62.5 Gy D 0.5 cc < 39 Gy D 30 cc < 35 Gy
Brachial plexus	D Max < 21 Gy	D Max < 30 Gy	D Max < 32 Gy
Trachea	D Max < 30 Gy	D Max < 32.5 Gy	Dmax < 46.3 Gy

**Table 3:** Patients and primary disease characteristics.

Age		
Mean	61 (Range 32–87)	
**Performance Status (ECOG)**	*n*	%
0	40	63%
1	21	33%
≥2	3	5%
**Histology**	*n*.	%
Ductal infiltrating carcinoma	53	83%
Lobular infiltrating carcinoma	5	8%
Other	6	9%
**Molecular classification**	n.	%
Luminal A	18	28%
Luminal B	18	28%
HER2 enriched	14	22%
Triple negative	13	20%

**Table 4:** Metastatic disease and SBRT characteristics.

Disease-Free Interval (Years)		
Mean (Range)	4.66 (0–17.8)	
**Type of metastatic disease**	n.	%
Synchronous	15	23%
Metachronous	49	77%
**Oligometastatic status at onset of disease**	n.	%
No	59	92%
Yes	5	8%
**Previous local ablative treatments (LAT)**	n.	%
No	44	69%
Yes	20	31%
**Lines of systemic therapies before SBRT**	n.	%
0	5	8%
1	23	36%
2	13	20%
≥3	23	36%
**Type of oligometastases**	n.	%
De-novo	15	23%
Repeat	6	10%
Induced	43	67%
**n. of radiated lesions**	n.	%
1	44	69%
2	15	23%
≥3	5	8%
**Organs receiving SBRT**	n.	%
Lung	23	36%
Liver	40	63%
Both	1	2%
**Number of organs receiving SBRT**	n.	%
1	63	98%
2	1	2%
**Disease extra SBRT target**	n.	%
Yes	23	36%
No	41	64%
**Concomitant systemic therapy**	n.	%
Yes	54	84%
No	10	16%
**BED**		
Mean	139.983	
Max	262.5	
Min	100	
Total dose/number of fractions	n. of lesions	%
45 Gy/3	10	11%
48 Gy/3	9	9.9%
54 Gy/3	3	3.3%
56.25 Gy/3	5	5.5%
60 Gy/3	13	14.3%
61.89 Gy/3	9	9.9%
67.5 Gy/3	1	1.1%
75 Gy/3	4	4.4%
48 Gy/4	15	16.5%
50 Gy/5	7	7.7%
54 Gy/6	3	3.3%
60 Gy/6	3	3.3%
63 Gy/6	3	3.3%
60 Gy/8	6	6.5%
**Subsequent systemic therapy**	n.	%
Yes	41	64%
No	23	36%

**Table 5:** Acute and late toxicities.

Toxicity			
Type		n.	%
**Acute toxicity**		15	23%
**G1**	-Fatigue	7	11%
	-Nausea and vomiting	4	6%
	-Fever	1	2%
	-Chest pain	1	2%
	-Abdominal pain	3	5%
	-Malaise	1	2%
**G2**	-Nausea and vomiting	2	3%
**Late toxicity**		9	14%
**G1**	-Chest pain	2	3%
	-Chough	1	2%
	-Pneumonia	1	2%
	-Rib fracture	1	2%
	-Gastritis	1	2%
	-Gastrointestinal pain	1	2%
**G2**	-Erythema	1	2%
	-Duodenal ulcer	1	2%

## Data Availability

Research data are stored in an institutional repository and will be shared upon request to the corresponding author.
